# Gated audiovisual speech identification in silence vs. noise: effects on time and accuracy

**DOI:** 10.3389/fpsyg.2013.00359

**Published:** 2013-06-19

**Authors:** Shahram Moradi, Björn Lidestam, Jerker Rönnberg

**Affiliations:** ^1^Linnaeus Centre HEAD, Department of Behavioral Sciences and Learning, Linköping UniversityLinköping, Sweden; ^2^Department of Behavioral Sciences and Learning, Linköping UniversityLinköping, Sweden

**Keywords:** audiovisual identification, gating paradigm, consonant, word, final word in sentences, silence, noise

## Abstract

This study investigated the degree to which audiovisual presentation (compared to auditory-only presentation) affected isolation point (IPs, the amount of time required for the correct identification of speech stimuli using a gating paradigm) in silence and noise conditions. The study expanded on the findings of Moradi et al. (under revision), using the same stimuli, but presented in an audiovisual instead of an auditory-only manner. The results showed that noise impeded the identification of consonants and words (i.e., delayed IPs and lowered accuracy), but not the identification of final words in sentences. In comparison with the previous study by Moradi et al., it can be concluded that the provision of visual cues expedited IPs and increased the accuracy of speech stimuli identification in both silence and noise. The implication of the results is discussed in terms of models for speech understanding.

## Introduction

The processing of spoken stimuli is interactive. Feed-forward from an incoming signal interacts with feedback from phonological representations in the mental lexicon for the identification of target signals (for a recent review, see Zion Golumbic et al., 2012). For audiovisual speech stimuli, there is additional processing between the incoming auditory and visual signals (see Besle et al., 2008; Lee and Noppeney, 2011). This forms a unified feed-forward signal that interacts with feedback from phonological representations in the mental lexicon [cf. Rapid Automatic Multimodal Binding of PHOnology [RAMBPHO] in the Ease of Language Understanding (ELU) model, Rönnberg et al., 2008]. The multiple interactive processing of audiovisual stimuli results in rapid and highly accurate identification compared with auditory or visual speech alone (Grant et al., 1998). Especially under degraded listening conditions, listeners tend to focus more on the movements of the speaker's face (Buchan et al., 2008). This partially protects the target signal from interference due to acoustic noise by providing information about when and where to expect an auditory signal (Grant, 2001), even though some phonemes and their features may not be readily extractable by vision.

## Audiovisual identification of consonants

Auditory cues provide information about the manner of articulation and voicing, whereas visual cues provide information about the place of articulation (Walden et al., 1975). Correspondence between auditory and visual articulation of phonemes is not one-to-one. Some consonants look the same during visual articulation, such as /k g η/ or /f v/. For instance, the auditory articulation of /b/ results in a clear perception of /b/ in optimum listening condition, while its visual correlates (or visemes) comprise the visual articulation for bilabial consonants /b p m/. The time at which auditory and visual modalities are accessed differs during the audiovisual identification of consonants (Munhall and Tohkura, 1998). Visual information is often available earlier than auditory information (Smeele, 1994).

The audiovisual identification of consonants occurs faster and is more accurate than unimodal auditory or visual presentation (Fort et al., 2010). This is probably due to the accessibility of complementary features associated with using both auditory and visual modalities. van Wassenhove et al. (2005) found that audiovisual speech was processed more quickly than auditory-alone speech. This rapid process was dependent on the degree of visibility of a speech signal; the process was more rapid for highly visible consonants, such as /pa/, than for less visible consonants, such as /ka/. van Wassenhove et al. (2005) proposed an on-line prediction hypothesis to explain how visual and auditory inputs might be combined during the audiovisual identification of speech stimuli. According to their hypothesis, initial visual input first activates phonological representations, and a prediction regarding the identity of the signal is made. This prediction is consistently updated with increasing visual input, and comparisons are made with auditory input in order to solve the identity of a signal. According to Grant and colleagues (Grant and Walden, 1996; Grant et al., 1998), there is little advantage to audiovisual presentation over unimodal presentation if the auditory and visual modalities provide the same critical features, whereas there is a greater advantage when each modality provides different critical features. The greatest advantage of the audiovisual presentation of consonants occurs when the stimuli are presented under noisy conditions (Grant et al., 1998; Jesse and Janse, 2012). Acoustically confusable phoneme pairs, such as /p/ and /k/, can be disambiguated using visual cues (Massaro and Stork, 1998). To conclude, the audiovisual identification of consonants is generally quicker than auditory-alone or visual-alone. As the phonetic cues from either modality act as predictors for phonetic cues from another modality, more rapid identification of audiovisual presentation would occur than unimodal presentations.

## Audiovisual identification of words

Word identification requires an association between an acoustic signal and the phonological-lexical representation in long-term memory (Rönnberg et al., 2008). In the audiovisual identification of words, information from both modalities is combined over time (Tye-Murray et al., 2007), resulting in faster and more accurate identification compared with auditory or visual stimuli alone (Fort et al., 2010). Tye-Murray et al. (2007) proposed the existence of audiovisual neighborhoods composed of overlaps between auditory and visual neighborhoods. According to this view, fewer words exist in the overlap between auditory and visual neighborhoods, resulting in the faster and more accurate identification of audiovisual words. Moreover, the information needed for the identification of vowels, which are the main constituents of words, is available earlier in visual than auditory signals (approximately 160 ms before the acoustic onset of the vowel; Cathiard et al., 1995). In addition, many words are only distinguishable by the place of articulation of one of their constituents (e.g., pet vs. net; Greenberg, 2005). The advantage of audiovisual word identification is more evident under noisy conditions (Sumby and Pollack, 1954; Kaiser et al., 2003; Sommers et al., 2005). Sumby and Pollack (1954) reported that 5–22 dB SNR more noise was tolerated in audiovisual presentation compared to auditory-alone presentation.

## Comprehension of audiovisual sentences

In the audiovisual identification of sentences, listeners can benefit from both contextual information and visual cues, resulting in the faster and more accurate identification of target words, especially under degraded listening conditions. The predictability level of sentences is a key factor (Conway et al., 2010); when the auditory signal is degraded, listeners exhibit better performance with highly predictable (HP) audiovisual sentences than with less predictable (LP) ones (Gordon and Allen, 2009). Grant and Seitz (2000) reported that spoken sentences masked by acoustic white noise were recognizable at a lower signal-to-noise ratio (SNR) when the speaker's face was visible. MacLeod and Summerfield (1987, 1990) showed that the provision of visual cues reduced the perceived background noise level by approximately 7–10 dB.

## Cognitive demands of audiovisual speech perception

Working memory acts as an interface between the incoming signal and phonological representations in semantic long-term memory (Rönnberg et al., 2008). According to the ELU model (Rönnberg et al., 2008), language understanding under optimum listening conditions for people with normal hearing acuity is mostly implicit and effortless. However, under degraded listening conditions (i.e., speech perception in background noise), the demand on the working memory system (including attention and inference-making skills) is increased to help disambiguate the impoverished acoustic signal and match it with corresponding phonological representations in semantic long-term memory. Support for this model comes from studies which show that language understanding under degraded listening conditions is cognitively taxing (for reviews see Rönnberg et al., 2010; Mattys et al., 2012). A recent neuroimaging study demonstrated increased functional connectivity between the auditory (middle temporal gyrus) and inferior frontal gyrus cortices during the perception of auditory speech stimuli in noise (Zekveld et al., 2012; see also Wild et al., 2012), thus suggesting an auditory–cognitive interaction.

Our previous study (Moradi et al., under revision) was in agreement with the ELU model's prediction. The findings showed that working memory and attentional capacities were positively correlated with the early correct identification of consonants and words in noise, while no correlations were found between the cognitive tests and identification of speech tasks in silence. In the noisy condition, listeners presumably are more dependent on their cognitive resources for keeping in mind, testing, and retesting hypothesis. In sum, a combination of auditory and explicit cognitive resources are required in speech perception, but to a lesser extent in silence than in noise.

Adding visual cues to the auditory signal may reduce the working memory load for the processing of audiovisual speech signals for the aforementioned reasons, and there are data to support this (Mousavi et al., 1995; Quail et al., 2009; Brault et al., 2010; Frtusova et al., 2013). Neuroimaging studies have shown that the superior temporal sulcus plays a critical role in audiovisual speech perception in both optimum and degraded listening conditions (Nath and Beauchamp, 2011; Schepers et al., 2013). For instance, Schepers et al. (2013) investigated how auditory noise impacts audiovisual speech processing at three different noise levels (silence, low, and high). Their results showed that auditory noise impacts on the processing of audiovisual speech stimuli in the lateral temporal lobe, encompassing the superior and middle temporal gyri. Visual cues precede auditory information because of natural coarticulatory anticipation, which results in a reduction in signal uncertainty and in the computational demands on brain areas involved in auditory perception (Besle et al., 2004). Visual cues also increase the speed of neural processing in auditory cortices (van Wassenhove et al., 2005; Winneke and Phillips, 2011). Audiological studies have shown that visual speech reduces the auditory detection threshold for concurrent speech sounds (e.g., Grant and Seitz, 2000). This reduction in the auditory threshold makes audiovisual stimuli much easier to detect, thereby reducing the need for explicit cognitive resources (e.g., working memory or attention). Pichora-Fuller (1996) presented sentences with and without background noise and measured the memory span of young adults. The results showed that subjects had better memory span in the audiovisual than in the auditory modality for sentences presented in noise.

Overall, the research indicates that audiovisual speech perception is faster, more accurate, and less effortful than auditory-alone or visual-alone speech perception. By inference, then, audiovisual speech will tax cognitive resources to a lesser extent than auditory-alone speech.

## Present study

This study is an extension of that by Moradi et al. (under revision); the same stimuli are used, but are instead presented audiovisually (as compared to auditory-only), using a different sample of participants. The study aimed to determine whether the added visual information would affect the amount of time required for the correct identification of consonants, words, and the final word of HP and LP sentences in both silence and noise using the gating paradigm (Grosjean, 1980). In the gating paradigm, participants hear and see successively increasing parts of speech stimuli until a target is correctly identified; the amount of time required for the correct identification of speech stimuli is termed the isolation point (IP). For example, the participant hears and sees the first 50 ms of a word, then the first 100 ms, and then the first 150 ms and so on, until he or she correctly identifies the word. The participant is required to speculate what the presented stimulus might be after each gate, and is usually also asked to give a confidence rating based on his or her guess. The IP is defined as the duration from the stimulus onset to the point at which correct identification is achieved and maintained without any change in decision after listening to the remainder of the stimulus (Grosjean, 1996).

### Predictions

We predicted that noise would delay the IPs and lower accuracy for the audiovisual identification of consonants and words, which is in line with the findings of our previous study (Moradi et al., under revision). For the audiovisual identification of final words in sentences, listeners can benefit from both the preceding context and visual cues; therefore, we predicted little or no effect of noise on the IPs and accuracy for final word identification in the audiovisual presentation of HP and LP sentences. We also expected that audiovisual presentation would be associated with faster IPs and better accuracy for all gated tasks, compared with auditory presentation alone [which was tested in Moradi et al. (under revision)]. Our previous study (Moradi et al., under revision) also demonstrated significant relationships between explicit cognitive resources (e.g., working memory and attention) and the IPs of consonants and words presented aurally in noise conditions. Specifically, better working memory and attention capacities were associated with the faster identification of consonants and words in noise. In contrast, in the present study, we predicted that the provision of visual cues would aid the identification of consonants and words in noise, and reduce the need for explicit cognitive resources. Hence, we predicted that there would be no significant correlations between the IPs of audiovisual speech tasks in noise and working memory and attention tasks in the present study.

## Methods

### Participants

Twenty-four participants (11 men, 13 women) were recruited from the student population of Linköping University. Their ages ranged from 19 to 32 years (*M* = 23.3 years). The students were monolingual Swedish native speakers. All reported having normal hearing and vision (or corrected-to-normal vision), with no psychological or neurological pathology. The participants received 500 SEK (Swedish Kronor) in return for their participation and provided written consent in accordance with the guidelines of the Swedish Research Council, the Regional Ethics Board in Linköping, and the Swedish practice for research on normal populations. It should be noted here that the group of participants in the present study did not differ in their characteristics (i.e., age, gender, educational level, vision and hearing status) with the group of Moradi et al. (under revision).

## Measures

### Gated speech tasks

A female native speaker of Swedish, looking directly into the camera, read all of the items at a natural articulation rate in a quiet studio. The hair, face, and top part of the speaker's shoulders were visible. She was instructed to begin each utterance with her mouth closed and to avoid blinking while pronouncing the stimuli. Visual recordings were obtained with a RED ONE digital camera (RED Digital Cinema Camera Company, CA) at a rate of 120 frames per second (each frame = 8.33 ms), in 2048 × 1536 pixels. The video recording was edited into separate clips of target stimuli so that the start and end frames of each clip showed a still face.

The auditory stimuli were recorded with a directional electret condenser stereo microphone at 16 bits, with a sampling rate of 48 kHz. The onset time of each auditory target was located as precisely as possible by inspecting the speech waveform using Sound Studio 4 (Felt Tip Inc., NY). Each segmented section was then edited, verified, and saved as a “.wav” file. The root mean square amplitude was computed for each stimulus waveform, and the stimuli were then rescaled to equalize amplitude levels across the different stimuli. A steady-state white noise, borrowed from Hällgren et al. (2006), was resampled and spectrally matched to the speech signals for use as background noise.

#### Consonants

Eighteen Swedish consonants were used, structured in vowel-consonant-vowel syllable format (/aba, ada, afa, aga, aja, aha, aka, ala, ama, ana, aŋa, apa, ara, aʈa, asa, aʃa, ata, and ava/). The gate size for consonants was set at 16.67 ms. The gating started after the first vowel, /a/, immediately at the start of the consonant onset. Thus, the first gate included the vowel /a/ plus the initial 16.67 ms of the consonant, the second gate added a further 16.67 ms of the consonant (total of 33.33 ms), and so on. The consonant-gating task took 25–40 min per participant to complete.

#### Words

The words in this study were in consonant-vowel-consonant format, chosen from a pool of Swedish monosyllabic words. The selected words had average to high frequencies according to the Swedish language corpus PAROLE 2011). In total, 46 words were chosen; these were divided into two lists (A and B), each containing 23 words. Both lists were matched in terms of onset phonemes and frequency of use in the Swedish language according to PAROLE (more specifically, each word had three to six alternative words with the same format and pronunciation of the first two phonemes, e.g., the target word /dop/ had the neighbors /dog, dok, don, dos/). For each participant, we presented one list in the silence condition and the other in the noise condition. The sequence of words was randomized across participants. A pilot study showed that the gate size used for consonants (16.67 ms) led to the subjective feeling that the word-identification task was monotonous, resulting in fatigue and loss of motivation. Therefore, a doubled gate size of 33.3 ms was used for word identification. The first phoneme (consonant) of each word was presented as a whole, and gating was started at the onset of the second phoneme (vowel). The word-gating task took 35–40 min per participant to complete.

#### Final words in sentences

This study compromised two types of sentences: HP and LP sentences. Predictability was categorized according to the last target word in each sentence which was always a monosyllabic noun (e.g., “Lisa gick till biblioteket för att låna en *bok*”; [Lisa went to the library to borrow a *book*] for an HP sentence; and “Färgen på hans skjorta var *vit*,” [The color of his shirt was *white*] for an LP sentence). The predictability of each target word, which was determined on the basis of the preceding words in the sentence, had been assessed in a previous pilot study (Moradi et al., under revision). There were 44 sentences: 22 in each of the HP and LP conditions. The gating started at the onset of the first phoneme of the target word. Due to the supportive effects of the context on word recognition, and based on the pilot data, we set the gate size at 16.67 ms to optimize resolution time. The sentence-gating task took 25–35 min per participant to complete.

### Hearing in noise test (HINT)

A Swedish version of the Hearing in Noise Test (HINT) (Hällgren et al., 2006), adapted from Nilsson et al. (1994), was used to measure the hearing-in-noise ability of the participant. The HINT sentences consisted of three to seven words. The participants had to repeat each entire sentence correctly in an adaptive ±2 dB SNR. That is, a correct response was followed by a decrease in SNR by 2 dB, and an incorrect response by an increase in SNR by 2 dB. The dependent measure is the calculated SNR (in our case for 50% correct performance). The HINT took approximately 10 min per participant to complete.

### Cognitive tests

#### Reading span test

In the reading span test (Baddeley et al., 1985), sentences were presented visually, word-by-word in the middle of a computer screen. After each sentence, the participants were instructed to determine whether the sentence was semantically correct or not. After the presentation of a set of sentences, the participants were instructed to repeat either the first word or the last word of each sentence, in correct serial order. Half of the sentences were semantically incorrect, and the other half were semantically correct (Rönnberg, 1990). In this study, two sets of three sentences were initially presented, then two sets of four sentences, followed by two sets of five sentences (for a total of 24 sentences). The reading span score was the aggregated number of words that were correctly recalled across all sentences in the test (maximum score = 24). The reading span test took approximately 15 min per participant to complete.

#### Paced auditory serial addition test (PASAT)

The PASAT is a test of executive functioning with a strong component of attention (Tombaugh, 2006). The task requires subjects to attend to auditory input, to respond verbally, and to inhibit the encoding of their responses, while simultaneously attending to the next stimulus in a series. Participants were presented with a random series of audio recordings of digits (1–9) and instructed to add pairs of numbers so that each number was added to the number immediately preceding it. This study used the PASAT 2 and PASAT 3 versions of the test (Rao et al., 1991), in which digits were presented at intervals of 2 or 3 s, respectively. The experimenter presented written instructions on how to complete the task, and each participant performed a practice trial. Participants started with PASAT 3, followed by PASAT 2 (faster rate), with a short break between the two tests. The total number of correct responses (maximum possible = 60) at each pace was recorded. The PASAT took approximately 10 min per participant to complete.

### Signal-to-noise ratio (SNR)

In our previous auditory gating study (Moradi et al., under revision), we adjusted the difference between signal and noise to 0 dB. A pilot study for the previous study revealed that very low SNRs resulted in too many errors and SNRs higher than 0 dB were too easy for identification. As the present study was interested in comparing the audiovisual findings with the auditory findings of our previous study (Moradi et al., under revision), we again set the SNR to 0 dB for all audiovisual stimuli.

## Procedure

Stimuli were synchronized within 1 ms accuracy and presented using MATLAB (R2009b) and Psychophysics Toolbox (version 3) on an Apple Macintosh computer (Mac Pro 4.1) running OS X (version 10.6.8) (cf. Lidestam, under revision, for more details). The computer was equipped with a fast solid-state hard drive and a fast interface (SATA-III, 6 Gb/s) and graphic card (ATI Radeon HD, 4870 GHz) to assure adequate speed for video rendering and playback. Visual stimuli were displayed in 600 × 600 pixels on a 22″ CRT monitor (Mitsubishi Diamond Pro 2070SB, 120-Hz refresh rate, 800 × 600-pixel resolution) and viewed from a distance of 55 cm. Audio signals were presented binaurally at approximately 65 dB (the range was 62.5–67 dB) via headphones (Sennheiser HDA200), having been adjusted to a comfortable level following the procedure in Moradi et al. (under revision). A second monitor was used for the setup of the experiment; this displayed the MATLAB script and enabled the experimenter to monitor the participants' progress. A screen was placed between the stimulus presentation monitor and the second monitor, preventing participants from seeing the experimenter's screen and response sheets.

The participants were tested individually in a quiet room. Each participant completed all of the gated tasks (consonants, words, and sentences) in one session (the first session), with short rest periods to prevent fatigue. All participants started with the identification of consonants, followed by words and then sentences. The type of listening condition (silence or noise) was counterbalanced across participants such that half of the participants started with consonant identification in the silence condition, and then proceeded to consonant identification in the noise condition (and vice versa for the other half of the participants). The order of items within each group of consonants, words, and sentences was randomized between participants. The participants were instructed to attend to the auditory speech and the speaker's face on-screen. The participants received written instructions about how to perform the gated tasks, how many sets there were in silence and noise, respectively, and completed several practice trials prior to the main task session. Participants were told to attempt identification after each presentation, regardless of how unsure they were about their identification of the stimulus, but to avoid random guessing. Participants gave their responses aloud, and the experimenter recorded the responses. When necessary, the participants were asked to clarify their responses. The presentation of gates continued until the target was correctly identified on six consecutive presentations. If the target was not correctly identified, stimulus presentation continued until the entire target was presented, even if six or more consecutive responses were identical. The experimenter then started the next trial. When a target was not identified correctly, even after the whole target had been presented, its total duration plus one gate size was used as the estimated IP (cf. Walley et al., 1995; Metsala, 1997; Hardison, 2005). The rationale for this calculated IP was the fact that it is possible some participants give their correct responses at the last gate of a given signal. Hence, estimating an IP equal to the total duration of that speech signal for both correct (even when late) and wrong responses would not be appropriate^1^. There was no specific feedback at any time during the session, except for general encouragement. Furthermore, there was no time pressure for responding to what was heard. The full battery of gating tasks took 85–110 min per participant to complete.

In the second session, the HINT, the reading span test, and the PASAT were administered. The order of the tests was counterbalanced across the participants. The second session took approximately 40 min per participant to complete.

## Design

The overall design for the gated tasks, which includes the comparative data from the Moradi et al. (under revision) study, was a 2 × 2 × 4 split-plot factorial design, with Modality as a between participants variable (audiovisual, auditory), combined with the within participant variables: Listening Condition (silence, noise) and Task (consonants, words, LP sentences, HP sentences). For the analysis of the consonant gating task, the design was 2 × 2 ×18 split-plot factorial: Modality × Listening Condition × Consonant. For the analysis of the word gating task, the design was 2 × 2 split-plot factorial: Modality × Listening Condition. For the final-word-in-sentence gating task, the design was 2 × 2 × 2 split-plot factorial: Modality × Listening Condition × Sentence Predictability.

## Results

### Gated audiovisual tasks

Table 1 reports the mean responses of participants for the HINT, PASAT 3, PASAT 2, and the reading span test for both the present study and that of Moradi et al. (under revision). There were no significant differences between the two studies for the PASAT 3, PASAT 2, and the reading span test scores. However, the HINT performance was significantly better in the present study than in Moradi et al. (under revision).

**Table 1:** **Means, *SD* (in parentheses), and significance levels for the HINT and cognitive tests in the present study and in Moradi et al. (under revision)**.

**Type of task**	**Mean (*SD*) in the present study**	**Mean (*SD*) in Moradi et al. (under revision)**	***p***
HINT	−4.17 (0.72)	−3.11 (1.22)	0.001
PASAT 3	53.38 (4.85)	51.19 (4.38)	0.122
PASAT 2	41.21 (8.33)	40.05 (6.16)	0.602
Reading span test	22.25 (1.67)	21.62 (1.69)	0.216

**Figure 1:**
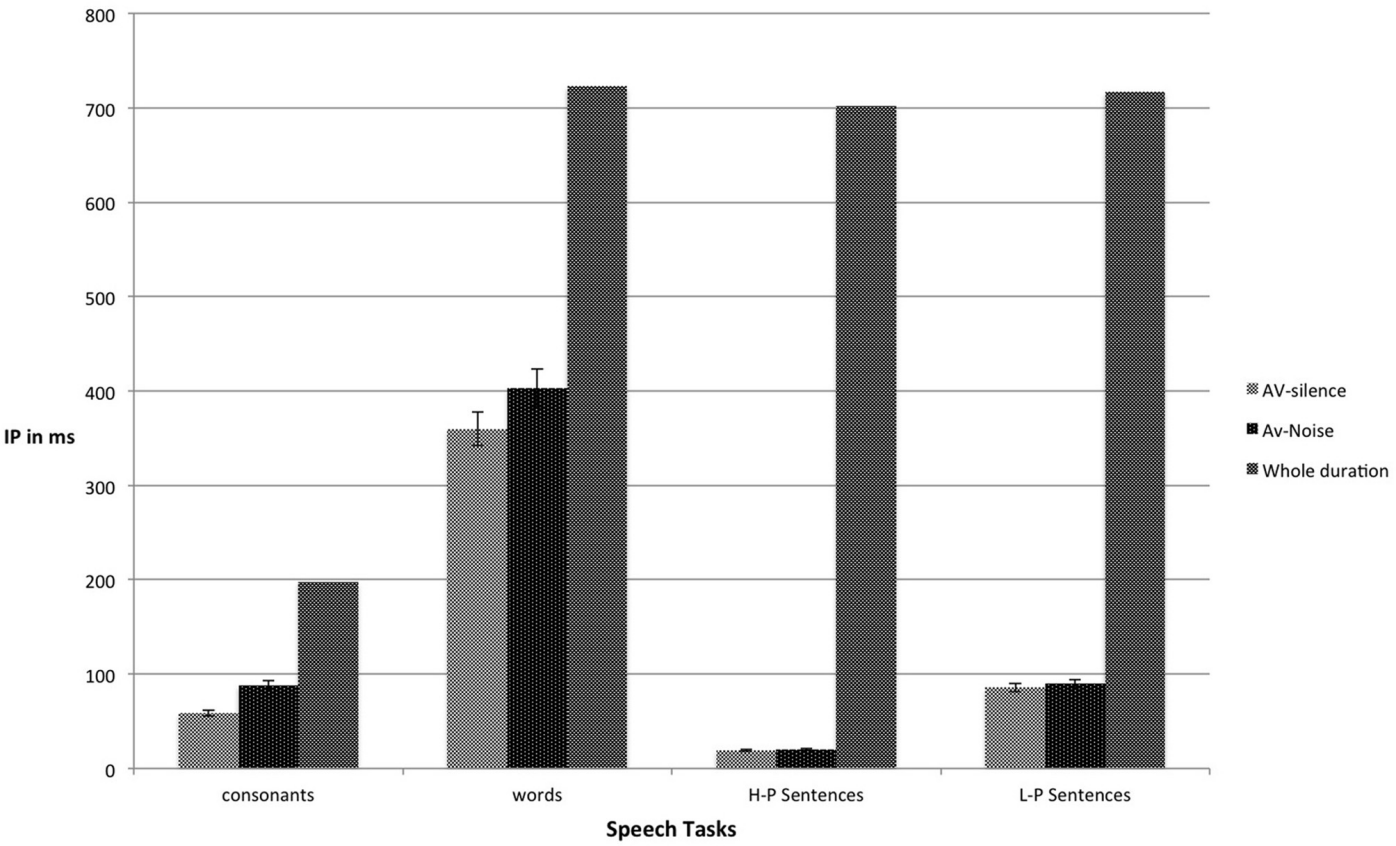
**Mean IPs (ms), with accompanying standard errors, for correct identification of audiovisual consonants, words, and final words in HP and LP sentences, in both silence and noise**. Whole duration refers to the average total duration from onset to offset.

Figure 1 shows the mean IPs for the audiovisual gated tasks in both the silence and noise conditions. A two-way repeated-measures analysis (ANOVA) was conducted to compare the means IP for each of the four gated tasks in silence and noise. The results showed a main effect of listening condition, *F*_(1, 23)_ = 50.69, *p* < 0.001, η^2^_*p*_ = 0.69, a main effect of the gated tasks, *F*_(1.78, 40.91)_ = 2898.88, *p* < 0.001, η^2^_*p*_ = 0.99, and an interaction between listening condition and gated tasks, *F*_(3, 69)_ = 17.57, *p* < 0.001, η^2^_*p*_ = 0.43. Four planned comparisons showed that the mean IPs of consonants in silence occurred earlier than in noise, *t*_(23)_ = 6.77, *p* < 0.001. In addition, the mean IPs of words in silence occurred earlier than in noise, *t*_(23)_ = 6.09, *p* < 0.001. However, the mean IPs of final words in HP sentences in silence did not occur earlier than in noise, *t*_(23)_ = 0.74, *p* > 0.05. The same was true for the mean IPs of final words in LP sentences, *t*_(23)_ = 0.76, *p* > 0.05.

Table 2 shows the mean number of correct responses for each of the gated tasks in the silence and noise presented in the audiovisual and auditory modalities. A 2 (Modality: audiovisual vs. auditory) × 2 (Listening Condition: silence vs. noise) × 4 (Gated Task: consonants, words, final words in HP and LP sentences) mixed ANOVA with repeated measures on the second and third factors was conducted to examine the effect of presentation modality on the accuracy for each of four gated tasks. The results showed a main effect of modality, *F*_(1, 43)_ = 275.32, *p* < 0.001, η^2^_*p*_ = 0.87, a main effect of listening condition, *F*_(1, 43)_ = 286.85, *p* < 0.001, η^2^_*p*_ = 0.87, a main effect of the gated tasks, *F*_(3, 129)_ = 38.15, *p* < 0.001, η^2^_*p*_ = 0.47, an interaction between presentation modality and the gated tasks, *F*_(3, 129)_ = 31.17, *p* < 0.001, η^2^_*p*_ = 0.42, an interaction between presentation modality and listening condition, *F*_(1, 43)_ = 145.83, *p* < 0.001, η^2^_*p*_ = 0.77, and a three-way interaction between modality, listening condition, and the gated tasks, *F*_(3, 129)_ = 26.27, *p* < 0.001, η^2^_*p*_ = 0.38. When comparing the accuracy of audiovisual relative to auditory presentation, the greatest advantage of audiovisual presentation was observed for word identification in noise. In the audiovisual modality, noise reduced the accuracy for consonants and words, whereas no effect of noise was found for the accuracy of final words in HP and LP sentences. In the auditory modality, noise reduced the accuracy for all of gated speech tasks. In addition, the most effect of noise on the accuracy in the auditory modality was observed for word identification.

**Table 2:** **Accuracy percentages for the identification of gated audiovisual and auditory stimuli: Mean and *SD* (in parentheses)**.

	**Descriptive statistics**				**Inferential statistics**			
	**Audiovisual**		**Auditory**		**Audiovisual vs. auditory**		**Silence vs. noise**	
**Types of gated tasks**	**Listening condition**				**Silence (*df* = 43)**	**Noise (*df* = 43)**	**Audiovisual (*df* = 23)**	**Auditory (*df* = 20)**
	**Silence (a)**	**Noise (b)**	**Silence (c)**	**Noise (d)**	**(a–c)**	**(b–d)**	**(a–b)**	**(c–d)**
Consonants	99.54 (1.58)	89.12 (10.16)	97.35 (3.78)	70.11 (17.52)	*t* = 2.59, *p* < 0.013, *d* = 0.76	*t* = 4.52, *p* < 0.001, *d* = 1.33	*t* = 4.85, *p* < 0.001, *d* = 1.37	*t* = 7.50, *p* < 0.001, *d* = 2.21
Words	100 (0.0)	93.84 (6.77)	96.27 (5.20)	34.58 (17.14)	*t* = 3.52, *p* < 0.001, *d* = 1.01	*t* = 15.62, *p* < 0.001, *d* = 4.55	*t* = 4.45, *p* < 0.001, *d* = 0.91	*t* = 15.14, *p* < 0.001, *d* = 4.26
Final words in LP	100 (0.0)	96.38 (9.90)	87.30 (7.27)	67.06 (20.32)	*t* = 8.57, *p* < 0.001, *d* = 2.47	*t* = 6.27, *p* < 0.001, *d* = 1.83	*t* = 1.79, *p* > 0.05, *d* = 0.36	*t* = 4.28, *p* < 0.001, *d* = 1.10
Final words in HP	99.62 (1.86)	100 (0.0)	94.84 (7.67)	85.71 (7.97)	*t* = 2.96, *p* < 0.005, *d* = 0.86	*t* = 8.80, *p* < 0.001, *d* = 2.54	*t* = 1.00, *p* > 0.05, *d* = 0.20	*t* = 2.90, *p* < 0.009, *d* = 1.51

### Comparison between gated audiovisual and auditory tasks

The next step in the analysis was to compare the IPs of the audiovisual tasks in the present study with those observed in our previous study (Moradi et al., under revision). This comparison (see Table 3) enabled to investigation of the impact that the addition of visual cues had on the amount of time required for the correct identification of stimuli in the auditory gated speech tasks. A 2 (Modality: audiovisual vs. auditory) × 2 (Listening Condition: silence vs. noise) × 4 (Gated Task: consonants, words, final words in HP and LP sentences) mixed ANOVA with repeated measures on the second and third factors was computed to examine the effect of presentation modality on the mean IPs for each of four gated tasks. The results showed a main effect of modality, *F*_(1, 43)_ = 407.71, *p* < 0.001, η^2^_*p*_ = 0.90, a main effect of listening condition, *F*_(1, 43)_ = 282.70, *p* < 0.001, η^2^_*p*_ = 0.87, a main effect of the gated tasks, *F*_(2, 67)_ = 2518.60, *p* < 0.001, η^2^_*p*_ = 0.98, an interaction between presentation modality and the gated tasks, *F*_(3, 129)_ = 89.21, *p* < 0.001, η^2^_*p*_ = 0.68, an interaction between presentation modality and listening condition, *F*_(1, 43)_ = 149.36, *p* < 0.001, η^2^_*p*_ = 0.78, and a three-way interaction between modality, listening condition, and the gated tasks, *F*_(3, 41)_ = 40.84, *p* < 0.001, η^2^_*p*_ = 0.49. When comparing the IPs of audiovisual relative to auditory presentation, the greatest advantage of audiovisual presentation in the silence condition was observed for identification of consonants and words. In the noise condition, the greatest advantage was observed for word identification. Also, when comparing the IPs in the silence condition relative to in the noise condition, the most delaying effect of noise was observed for word identification in the auditory modality. In the audiovisual modality, noise effectively delayed identification of consonants and words, whereas no effect of noise was found for identification of final words in HP and LP sentences.

**Table 3:** **Descriptive and inferential statistics for ips of consonants, words, and final words in HP and LP sentences in silence and noise presented audiovisually and auditorily**.

	**Descriptive statistics**				**Inferential statistics**			
	**Audiovisual**		**Auditory**		**Audiovisual vs. auditory**		**Silence vs. noise**	
**Types of gated tasks**	**Listening condition**				**Silence (*df* = 43)**	**Noise (*df* = 43)**	**Audiovisual (*df* = 23)**	**Auditory (*df* = 20)**
	**Silence (a)**	**Noise (b)**	**Silence (c)**	**Noise (d)**	**(a–c)**	**(b–d)**	**(a–b)**	**(c–d)**
Consonants	58.46 (11.38)	85.01 (19.44)	101.78 (11.47)	161.63 (26.57)	*t* = 12.69, *p* < 0.001, *d* = 3.87	*t* = 11.14, *p* < 0.001, *d* = 3.40	*t* = 6.17, *p* < 0.001, *d* = 1.84	*t* = 12.02, *p* < 0.001, *d* = 3.15
Words	359.78 (25.97)	403.18 (32.06)	461.97 (28.08)	670.51 (37.64)	*t* = 12.68, *p* < 0.001, *d* = 3.87	*t* = 25.73, *p* < 0.001, *d* = 7.85	*t* = 6.09, *p* < 0.001, *d* = 1.49	*t* = 17.73, *p* < 0.001, *d* = 6.30
Final words in LP	85.68 (22.55)	89.94 (15.93)	124.99 (29.09)	305.18 (121.20)	*t* = 5.10, *p* < 0.001, *d* = 1.56	*t* = 8.63, *p* < 0.001, *d* = 2.63	*t* = 0.76, *p* > 0.05, *d* = 0.22	*t* = 7.67, *p* < 0.001, *d* = 2.04
Final words in HP	19.32 (2.69)	19.95 (3.84)	23.96 (3.31)	48.57 (23.01)	*t* = 5.18, *p* < 0.001, *d* = 1.58	*t* = 6.01, *p* < 0.001, *d* = 1.83	*t* = 0.74, *p* > 0.05, *d* = 0.19	*t* = 4.96, *p* < 0.001, *d* = 1.50

#### Consonants

**Figure 2:**
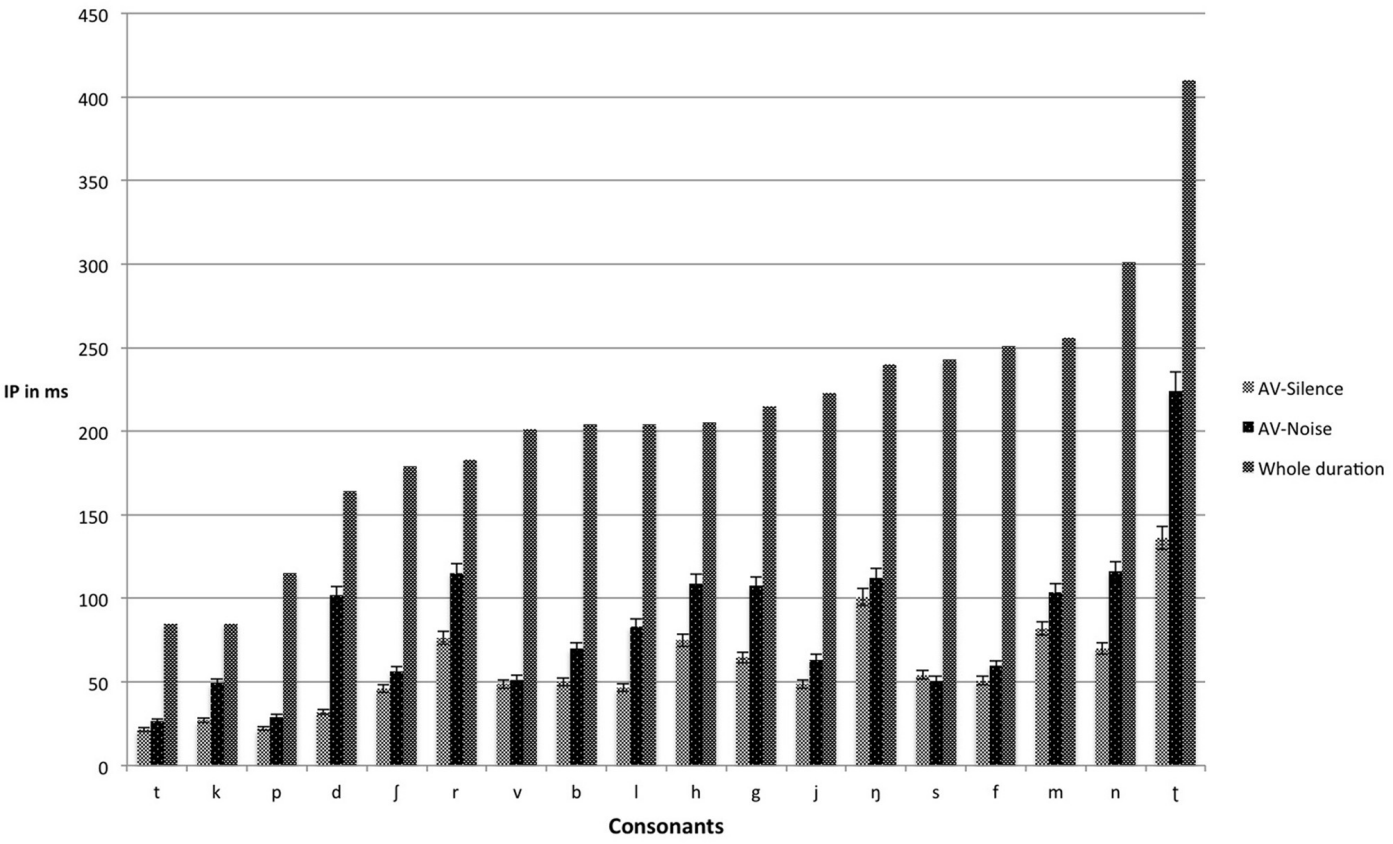
**Mean IPs (ms), with accompanying standard errors, for correct identification of audiovisual consonants in both silence and noise**. Whole duration refers to the total duration from onset to offset.

Table 4 shows the mean IPs for the correct identification of consonants in silence and noise presented in the audiovisual and auditory modalities (see also Figure 2 for the IPs of audiovisual consonants in silence and noise relative to their total durations). A 2 (Modality: audiovisual vs. auditory) × 2 (Listening Condition: silence vs. noise) × 18 (Consonants) mixed ANOVA with repeated measures on the second and third factors was conducted to examine the effect of presentation modality on the IPs for consonant identification. The results showed a main effect of modality, *F*_(1, 43)_ = 204.50, *p* < 0.001, η^2^_*p*_ = 0.83, a main effect of listening condition, *F*_(1, 41)_ = 174.09, *p* < 0.001, η^2^_*p*_ = 0.80, a main effect for consonants, *F*_(6, 273)_ = 61.16, *p* < 0.001, η^2^_*p*_ = 0.59, and a three-way interaction between modality, listening condition, and consonants, *F*_(17, 27)_ = 2.42, *p* < 0.001, η^2^_*p*_ = 0.05. Subsequent *t*-test comparisons using a Bonferroni adjustment revealed significant differences (*p* < 0.00278) between silence and noise for /*b f h j k l m n p r* ʃ *t v*/ within the auditory modality. However, except for /*d k*/, the addition of visual cues did not result in significant differences (*p* > 0.00278) between silence and noise for consonants presented audiovisually. The addition of visual cues did not significantly affect the IPs of /ŋ ʈ *g s*/ in neither silence nor noise, that is, there were no differences between the auditory and audiovisual modalities for these consonants.

**Table 4:** **Mean IPs, *SD* (in parentheses), and significance levels for the identification of consonants presented audiovisually and auditorily in silence and noise**.

	**Modality**				***p***			
**Consonants**	**Audiovisual**		**Auditory**		**Audiovisual vs. auditory**		**Silence vs. noise**	
	**Listening condition**				**Silence**	**Noise**	**Audiovisual**	**Auditory**
	**Silence (a)**	**Noise (b)**	**Silence (c)**	**Noise (d)**	**(a–c)**	**(b–d)**	**(a–b)**	**(c–d)**
*b*	50.01 (38.08)	70.15 (44.24)	89.70 (38.19)	157.97 (58.13)	**0.001**	**0.001**	0.069	**0.001**
*d*	31.96 (23.53)	102.10 (51.86)	138.92 (29.51)	158.76 (25.62)	**0.001**	**0.001**	**0.001**	0.025
*f*	50.70 (31.28)	59.73 (68.45)	86.53 (17.97)	178.61 (66.92)	**0.001**	**0.001**	0.425	0.001
*g*	64.60 (37.54)	107.66 (80.92)	146.06 (39.77)	183.37 (47.44)	**0.001**	**0.001**	0.022	0.018
*h*	75.02 (20.86)	109.05 (57.32)	96.05 (22.31)	186.55 (44.92)	**0.002**	**0.001**	0.007	**0.001**
*j*	48.62 (22.48)	63.21 (40.23)	66.68 (21.74)	130.18 (41.38)	0.009	**0.001**	0.112	**0.001**
*k*	27.09 (12.83)	49.32 (25.30)	54.77 (19.11)	85.73 (13.22)	**0.001**	**0.001**	**0.001**	**0.001**
*l*	46.54 (23.56)	83.35 (72.58)	84.94 (17.41)	176.23 (35.97)	**0.001**	**0.001**	0.014	**0.001**
*m*	81.96 (31.44)	103.49 (56.69)	79.38 (15.73)	148.44 (72.64)	0.735	0.025	0.044	**0.001**
*n*	70.15 (48.41)	116.00 (82.62)	105.58 (32.64)	199.25 (61.13)	0.007	**0.001**	0.016	**0.001**
ŋ	100.71 (42.99)	112.52 (72.79)	162.73 (52.16)	169.88 (65.34)	**0.001**	0.008	0.310	0.661
*p*	22.23 (13.61)	29.17 (26.13)	66.68 (14.91)	111.93 (16.79)	**0.001**	**0.001**	0.226	**0.001**
*r*	76.40 (25.03)	115.30 (55.38)	88.11 (23.66)	169.88 (34.82)	0.116	**0.001**	0.005	**0.001**
ʈ	136.14 (102.37)	224.35 (156.07)	231.00 (109.60)	338.96 (116.61)	0.004	0.009	0.033	0.008
*s*	54.18 (11.26)	50.70 (11.51)	68.27 (16.59)	103.99 (65.82)	**0.002**	**0.001**	0.307	0.017
ʃ	45.84 (17.21)	56.26 (43.36)	115.90 (31.84)	166.70 (50.84)	**0.001**	**0.001**	0.295	**0.001**
*t*	21.53 (10.40)	26.39 (14.68)	44.45 (19.25)	84.94 (13.85)	**0.001**	**0.001**	0.110	**0.001**
*v*	48.62 (36.10)	51.40 (45.83)	106.37 (47.87)	157.97 (43.67)	**0.001**	**0.001**	0.771	**0.001**

Significant differences according to Bonferroni adjustment (p < 0.00278) are in bold.

#### Words

A 2 (Modality: audiovisual vs. auditory) × 2 (Listening Condition: silence vs. noise) mixed ANOVA with repeated measures on the second factor was conducted to examine the effect of presentation modality on the IPs for word identification. The results showed a main effect of modality, *F*_(1, 43)_ = 818.21, *p* < 0.001, η^2^_*p*_ = 0.95, a main effect of listening condition, *F*_(1, 43)_ = 354.88, *p* < 0.001, η^2^_*p*_ = 0.89, and an interaction between modality and listening condition, *F*_(1, 43)_ = 152.47, *p* < 0.001, η^2^_*p*_ = 0.78. One-tailed *t*-tests were subsequently carried out to trace the source of interaction. The results showed that mean audiovisual word identification in silence occurred earlier than mean auditory word identification in silence, *t*_(43)_ = 12.68, *p* < 0.001. In addition, mean audiovisual word identification in noise was earlier than mean auditory word identification in noise, *t*_(43)_ = 25.73, *p* < 0.001. As Table 3 shows, the difference between silence and noise is larger in the auditory modality than in the audiovisual modality, indicating a less delaying effect of noise in the audiovisual modality.

#### Final words in sentences

A 2 (Modality: audiovisual vs. auditory) × 2 (Listening Condition: silence vs. noise) × 2 (Sentence Predictability: high vs. low) mixed ANOVA with repeated measures on the second and third factors was conducted to examine the effect of presentation modality on the IPs for final-word identification in sentences. The results showed a main effect of modality, *F*_(1, 43)_ = 79.68, *p* < 0.001, η^2^_*p*_ = 0.65, a main effect of listening condition, *F*_(1, 43)_ = 68.11, *p* < 0.001, η^2^_*p*_ = 0.61, and a main effect of sentence predictability, *F*_(1, 43)_ = 347.60, *p* < 0.001, η^2^_*p*_ = 0.89. There was a three-way interaction between modality, listening condition, and sentence predictability, *F*_(1, 43)_ = 53.32, *p* < 0.001, η^2^_*p*_ = 0.55. Subsequent one-tailed *t*-tests showed that the mean final word identification in both HP and LP sentences occurred earlier in the audiovisual than in the auditory presentation in both silence and noise. As Table 3 shows, the greatest advantage of audiovisual presentation was observed for final-word identification in LP sentences the in noise condition. In addition, when comparing IPs in silence relative to noise, the most delaying effect of noise was observed for final-word identification in LP sentences in the auditory modality.

### Correlations between audiovisual gated tasks, the HINT, and cognitive tests

Table 5 shows the Pearson correlations between the IPs for the different gated tasks (lower scores for the gated tasks reflect better performance), the HINT scores (lower scores for the HINT reflect better performance), and the reading span test and PASAT scores (higher scores for the reading span test and PASAT reflect better performance), in both listening conditions (silence and noise). The PASAT 2 was significantly correlated with the HINT and the reading span test. The reading span test was also significantly correlated with the HINT, PASAT 2, and PASAT 3. In addition, the HINT was significantly correlated with IPs of words in noise: the better the participants performed on the HINT, the earlier they could generally identify words presented in noise (and vice versa).

**Table 5:** **Correlations between IPs for the gated audiovisual speech tasks, the HINT, and the cognitive tests**.

	**1**	**2**	**3**	**4**	**5**	**6**	**7**	**8**	**9**	**10**	**11**	**12**
1. HINT		−0.34	−0.64^**^	−0.63^**^	0.29	0.15	0.12	0.42^*^	0.15	0.08	−0.04	0.09
2. PASAT 3			0.70^**^	0.48^*^	0.09	−0.06	0.01	−0.25	0.06	−0.38	−0.34	−0.07
3. PASAT 2				0.64^**^	−0.03	0.06	−0.32	−0.27	−0.13	−0.38	−0.15	−0.30
4. RST					−0.05	−0.14	−0.24	−0.40	−0.12	−0.10	0.23	−0.37
5. Consonant-S						0.14	0.09	−0.10	0.29	0.24	0.06	−0.14
6. Consonant-N							−0.34	−0.29	−0.13	−0.18	−0.17	−0.29
7. Word-S								0.29	0.27	0.30	−0.04	0.43
8. Word-N									0.20	0.01	−0.03	0.26
9. HP-S										0.41^*^	0.21	0.02
10. LP-S											0.54^**^	0.01
11. HP-N												−0.10
12. LP-N												

Notes: RST, Reading Span Test; Consonant-S, Gated consonant identification in silence; Consonant-N, Gated consonant identification in noise; Word-S, Gated word identification in silence; Word-N, Gated word identification in noise; HP-S, Gated final-word identification in highly predictable sentences in silence; LP-S, Gated final-word identification in less predictable sentences in silence; HP-N, Gated final-word identification in highly predictable sentences in noise; LP-N, Gated final-word identification in less predictable sentences in noise.

* p < 0.05,

** p < 0.01.

## Discussion

### IPs for the identification of consonants, words, and final words in LP and HP sentences

#### Consonants

The mean IPs for consonant identification occurred earlier in silence than in noise (~58 ms in silence vs. 88 ms in noise), indicating that noise delayed audiovisual consonant identification. In accordance with the timing hypothesis proposed by van Wassenhove et al. (2005), we hypothesized that background noise would impact on the auditory input of the audiovisual signal, which may make a match between the preceding visual information and the predicted auditory counterparts more difficult, resulting in higher residual errors than in the silence. The resolution of this non-match would require more time (compared with the silence condition) to correctly match the preceding visual signal with the corresponding auditory input. The present study demonstrated that the amount of time required for the correct identification of consonants was highly variable in both silence and noise (Figure 2). The correct identification of consonants was nearly 100% in silence and dropped to 89% in noise (Table 2). This is consistent with the findings of Beskow et al. (1997), who reported that listeners correctly identified 76% of Swedish consonants in +3 dB SNR. In sum, our results support our prediction that noise delays IPs and lowers accuracy for the audiovisual identification of consonants.

When comparing the results of consonant identification in the present study with those of Moradi et al. (under revision), it is evident that the provision of visual cues made consonant identification occur earlier in both silence and noise. The results shown in Table 4 demonstrate that the consonants with the most distinctive visual cues, such as /*b f l m p* ʃ *t v*/ (cf. Lidestam and Beskow, 2006), were more resistant to noise. However, the added visual cues had no effect on the IPs for /ŋ ʈ *g s*/. Lidestam and Beskow (2006) showed that /ʈ/ was associated with the least visual identification, and /ŋ/ was among the consonants with low identification scores. In terms of accuracy, the correct identification of consonants presented auditory in noise was ~70% (Moradi et al., under revision). In the current study, this increased to 89% for consonants presented audiovisually. Thus, our findings corroborated the findings of Fort et al. (2010), which showed that audiovisual presentation resulted in higher accuracy and faster identification of phonemes in noise. Our results are also in line with those of Grant and Walden (1996) and Grant et al. (1998) who reported that the visual cues do not need to be very distinctive, as long as they provide cues that are not available from the auditory signal alone, which means that audiovisual identification of consonants in noise is super-additive. In fact, attentional cueing via preceding visual signals provides information about where or when (or where *and* when) the target speech should occur in noisy conditions (Best et al., 2007), which in turn facilitates speech perception in degraded listening conditions. The results were as predicted: audiovisual presentation generally speeded up IPs and improved the accuracy of identified consonants (compared with auditory presentation), and noise generally delayed IPs and lowered accuracy.

#### Words

The mean IPs for audiovisual word identification in silence occurred earlier than in noise (~360 ms vs. 403 ms, respectively), which indicates that noise made audiovisual word identification occur later. Audiovisual word identification IPs in noise was correlated with HINT performance (Table 5), which indicates that those with a better ability to hear in noise (when not seeing the talker) were also able to identify audiovisual words in noise faster (i.e., when they could see the talker) or vice versa (i.e., those who identified audiovisually presented words in noise early were generally better at hearing in noise when not seeing the talker). Table 2 shows that the accuracy for correctly identified words in noise was 94%. Our results are in line with those of Ma et al. (2009), who reported the accuracy for word identification to be 90% at 0 dB SNR for monosyllabic English words. Our results are also consistent with the audiovisual gating results of de la Vaux and Massaro (2004), wherein correct word identification at the end of gates was 80% at about +1 dB SNR (they presented stimuli at a maximum of 80% of the total duration of the words). Our results support our prediction that noise delays IPs and reduces accuracy for the audiovisual identification of words.

When comparing the results of the word identification task in the present study with those of our previous study (Moradi et al., under revision), there is an interaction between listening conditions and presentation modality, wherein the impact of noise is reduced in the audiovisual relative to the auditory modality. Audiovisual presentation accelerated word identification to such a degree that the mean IP in audiovisual word identification in noise (403 ms) was less than the mean IP for auditory word identification in silence (462 ms). One explanation as to why auditory word identification takes longer than audiovisual word identification can be inferred from the findings of Jesse and Massaro (2010). They showed that visual speech information is generally fully available early on, whereas auditory speech information is accumulated over time. Hence, early visual speech cues lead to rapid audiovisual word identification. Furthermore, according to Tye-Murray et al. (2007), input received from both auditory and visual channels results in fewer neighborhood candidates (in the overlap of auditory and visual signals) for audiovisual word identification. Together, the results suggest that the time taken to eliminate unrelated candidates when attempting to match an incoming signal with a phonological representation in long-term memory is shorter for words presented audiovisually. This modality protects the speech percept against noise compared to auditory-only presentation. Our results, which showed that the addition of visual cues accelerated lexical access, are consistent with those of Barutchu et al. (2008), Brancazio (2004), and Fort et al. (2010). In our previous study (Moradi et al., under revision), the mean accuracy for word identification in noise was 35%. This increased to 94% in audiovisual word identification in noise in the present study. This result is in line with Potamianos et al. (2001) who reported that at −1.6 dB, the addition of visual cues resulted in 46% improvement in the intelligibility of words presented in noise. As predicted, the results showed that the audiovisual presentation of words resulted in earlier IPs and better accuracy for word identification compared with auditory presentation.

#### Final words in sentences

As the results show, there was no difference in IPs between silence and noise conditions for final-word identification in HP and LP sentences. The visual cues had a greater compensatory effect for the delay associated with noise than the sentence context had. It did not appear to matter whether the degraded final word was embedded within an HP or LP sentence. The findings are in line with our prediction that noise should not impact significantly on IPs or accuracy for final word identification in HP and LP sentences.

When comparing the results from the present study with those of our previous study (Moradi et al., under revision), The greatest benefit of audiovisual presentation was for LP sentences in noise condition. In sum, there was added benefit associated with the provision of visual cues and the preceding context for the early decoding of final words in audiovisual sentences in noise. The results were in line with our prediction that audiovisual presentation would result in earlier IPs and better accuracy for final word identification in HP and LP sentences compared with auditory-only presentation.

### Effect of modality on the HINT performance

It should be noted that there was a significant difference between the HINT performance in the present study and the HINT performance in the study by Moradi et al. (under revision). In both studies, we administered the gated tasks (presented auditory or audiovisually) in the first session and the HINT and cognitive tests in the second session. Audiovisually gated presentation thus seemed to improve HINT performance compared to auditory-only gated presentation. In a study by Bernstein et al. (2013), which examined the impact of audiovisual training on degraded perceptual learning of speech, subjects learned to form paired associations between vocoded spoken nonsense words and nonsense pictures. In one of their experiments, audiovisual training was compared with auditory-only training, and the results showed that, when tested in an auditory-only condition, the audiovisually trained group was better at correctly identifying consonants embedded in nonsense words than the auditory-only group. In other words, auditory-only perception was significantly better following audiovisual training than following auditory-only training. Rosenblum et al. (2007) studied how prior exposure to lip-reading impacts on later auditory speech-in-noise performance. They presented subjects with lip-reading stimuli from the same or a different talker and then measured the auditory speech-in-noise identification performance. The results showed that lip-reading the same talker prior to testing enhanced auditory speech-in-noise performance. Rosenblum et al. hypothesized that the derived amodal idiolectic information from the visual speech of a talker is used to ease auditory speech-in-noise perception. In our studies, the talkers in the gating paradigm and the HINT were not the same but were two different females. To account for this improved HINT performance after audiovisual gating compared to auditory gating, we hypothesize that the cross-modal facilitation, as observed in the HINT scores after audiovisual-gating tasks, can exist even with different talkers to boost the identification of auditory speech-in-noise. According to our findings, we extend the hypothesis by Rosenblum et al. to suggest that visual cues derived from a different talker can still be used to facilitate auditory speech-in-noise function. Further studies are required to see if this cross-modal facilitation from different talkers can be replicated.

### Cognitive demands of audiovisual speech perception

The current results showed no significant relationships between identification of different audiovisual gated stimuli and performance on cognitive tests, in neither silence nor noise, which supports our prediction that audiovisual speech perception is predominantly effortless. In fact, the audiovisually presentation of speech stimuli reduces working memory load (i.e., Pichora-Fuller, 1996; Frtusova et al., 2013) which in turn eases processing of stimuli especially in noisy condition.

The present study corroborates the findings of our previous study (Moradi et al., under revision) regarding the correlations between the HINT and cognitive tests, such that the HINT was significantly correlated with the reading span test and PASAT 2, suggesting that the subjects with greater hearing-in-noise function had better attention and working memory abilities. When comparing the results from the present study with those of Moradi et al. (under revision), it can be concluded that the identification of audiovisual stimuli (at an equal SNR) demanded less in terms of attention and working memory. This finding is consistent with Fraser et al. (2010), who showed that in the noise condition, speech perception was enhanced and subjectively less effortful for the audiovisual modality than the auditory modality at an equivalent SNR. This is in line with the general prediction made by the ELU model, which states that for relatively poor input signal conditions (i.e., comparing auditory with audiovisual conditions), dependence on working memory and other executive capacities will increase (Rönnberg et al., 2008). We assume that the SNR in the noise condition was not sufficiently demanding to require explicit cognitive resources for the identification of audiovisual speech stimuli in noise; the perceived audiovisual speech signal was well perceived despite the noise. In other words, the audiovisual presentation protected the speech percepts against the noise that has been proven to be an effective masker. It is, however, likely that lower SNRs would increase the demand for explicit cognitive resources.

Our results are not consistent with those of Picou et al. (2011), which showed that low working memory capacity was associated with relatively effortful audiovisual identification of stimuli in noise. It should be noted that Picou et al. (2011) set the SNRs individually for each participant (the audiovisual SNRs ranged from 0 dB to −4 dB, with an average of −2.15 dB across participants). Thus, their method was different to ours, because we used a constant SNR across participants (*SNR* = 0 dB). Hence, the audiovisual task in the noise condition was more difficult in the study of Picou et al. (2011) and probably more cognitively demanding than in our study. Working memory may have been required for the task in the Picou and colleagues' study in order to aid the identification of an impoverished audiovisual signal (cf. the ELU model, Rönnberg et al., 2008). Rudner et al. (2012) showed a significant relationship between working memory capacity and ratings of listening effort for speech perception in noise. Thus, in Picou and colleagues' study, participants with larger working memory capacity may have processed the impoverished audiovisual signal with less effort than those with lower working memory capacity.

One limitation of the present study is that the auditory and audiovisual data stem from different samples, which may raise concerns about potential between-subject sampling errors (although the recruitment and test procedures were identical in both studies). A within-subject design would allow more robust interpretations. Awaiting such an experimental replication, the pattern of results in the current and the previous study by Moradi et al. replicate other independent studies and make theoretical sense. In addition, we used the reading span test and the PASAT with the assumption that they measure amodal working memory and attention capacities of participants. However, there is a concern about the fact that audiovisual speech tasks and working memory (or attention) was measured separately. In order to draw stronger conclusions about the effect of audiovisual presentation on the working memory (or attention) capacity, a working memory (or attention) task using audiovisual speech stimuli (cf. Frtusova et al., 2013 or Pichora-Fuller, 1996) is proposed for future studies.

## Conclusions

Our results demonstrate that noise significantly delayed the IPs of audiovisually presented consonants and words. However, the IPs of final words in audiovisually presented sentences were not affected by noise, regardless of the sentence predictability level. This suggests that the combination of sentence context and a speech signal with early visual cues resulted in fast and robust lexical activation. In addition, audiovisual presentation seemed to result in fast and robust lexical activation. Importantly, audiovisual presentation resulted in faster and more accurate identification of gated speech stimuli compared to an auditory-only presentation (Moradi et al., under revision).

### Conflict of interest statement

The authors declare that the research was conducted in the absence of any commercial or financial relationships that could be construed as a potential conflict of interest.

## Footnotes

^1^Similar to Metsala (1997), we also analyzed our data by only including correct responses. There was a main effect of modality, *F*_(1, 43)_ = 433.41, *p* < 0.001, η^2^_*p*_ = 0.91; a main effect of listening condition, *F*_(1, 43)_ = 55.38, *p* < 0.001, η^2^_*p*_ = 0.56; a main effect of gated tasks, *F*_(2, 76)_ = 8395.20, *p* < 0.001, η^2^_*p*_ = 0.99; an interaction between presentation modality and gated tasks, *F*_(3, 129)_ = 108.60, *p* < 0.001, η^2^_*p*_ = 0.72; and an interaction between presentation modality and listening condition, *F*_(1, 43)_ = 20.69, *p* < 0.001, η^2^_*p*_ = 0.33. However, the three-way interaction between modality, listening condition, and the gated tasks was not significant in this analysis, *F*_(3, 41)_ = 1.01, *p* > 0.05, η^2^_*p*_ = 0.02.
